# Degree of exercise intensity during continuous chest compression in upper-body-trained individuals

**DOI:** 10.1186/s40101-015-0079-x

**Published:** 2015-12-19

**Authors:** Hisayoshi Ogata, Ikuyo Fujimaru, Takaharu Kondo

**Affiliations:** Department of Lifelong Sports for Health, College of Life and Health Sciences, Chubu University, 1200 Matsumoto-cho, Kasugai, Aichi 487-8501 Japan

**Keywords:** Continuous chest compression, Upper body, Physical training, Fatigue

## Abstract

**Background:**

Although chest-compression-only cardiopulmonary resuscitation (CCO-CPR) is recommended for lay bystanders, fatigue is easily produced during CCO-CPR. If CCO-CPR can be performed at a lower intensity of exercise, higher resistance to fatigue is expected. Since chest compression is considered to be a submaximal upper body exercise in a steady rhythm and since the unit of load for chest compression is expressed as work rate, we investigated the possibility that peak work rate of the upper body determines the level of exercise intensity during CCO-CPR.

**Methods:**

Twelve sedentary individuals (group Se), 11 rugby players (group R), and 11 swimmers (group Sw) performed 10-min CCO-CPR, and heart rate (HR) and rating of perceived exertion (RPE) were measured as indices of exercise intensity. Multiple linear regression analysis was carried out to assess potential relationships of upper body weight, peak lumbar extension force, peak work rate, and peak oxygen uptake recorded during arm-crank exercise with HR and RPE during CCO-CPR.

**Results:**

Values of peak work rate during arm-crank exercise (Peak WR_-AC_) in group Se, group R, and group Sw were 108 ± 12, 139 ± 27, and 146 ± 24 watts, respectively. Values of the latter two groups were significantly higher than the value of group Se (group R, *P* < 0.01; group Sw, *P* < 0.001). HR during CCO-CPR increased with time, reaching 127.8 ± 17.6, 114.8 ± 16.5, and 118.1 ± 14.2 bpm at the 10th minute in group Se, group R, and group Sw, respectively. On the other hand, RPE during CCO-CPR increased with time, reaching 16.4 ± 1.4, 15.4 ± 1.7, and 13.9 ± 2.2 at the 10th minute in group Se, group R, and group Sw, respectively. Multiple linear regression analysis showed that only peak WR_-AC_ affects both HR and RPE at the 10th minute of CCO-CPR (HR, *r* = −0.458; *P* < 0.01; RPE, *r* = −0.384, *P* < 0.05).

**Conclusions:**

The degree of exercise intensity during CCO-CPR is lower in individuals who have a higher peak work rate of the upper body.

## Background

Cardiopulmonary resuscitation (CPR) by bystanders provides a favorable outcome after out-of-hospital cardiac arrest [1–6]. In countries such as Japan with an increasing proportion of elderly people in the population, it is expected that the number of elderly people with out-of-hospital cardiac arrest will increase [5]. Thus, the effectiveness of bystander CPR for elderly people [1, 5] is particularly important in an aging society. However, the low rate of bystander CPR is a major problem [7]. Bystanders would be more willing to perform chest-compression-only CPR (CCO-CPR) than conventional CPR consisting of chest compression (CC) and rescue breathing [8]. Thus, it is expected that the rate of bystander CPR could be improved by encouraging the use of CCO-CPR [9]. Indeed, a recent study has shown that dispatcher instruction of CCO-CPR increases actual provision of bystander CPR compared to dispatcher instruction of conventional CPR [10]. It has also been demonstrated that CCO-CPR was associated with a more favorable outcome than was conventional CPR [2, 6].

On the other hand, one important issue is that more fatigue is produced by CCO-CPR than by conventional CPR [11]. If CCO-CPR can be performed at a lower intensity of exercise, higher resistance to fatigue is expected [12]. It has been shown that heart rate (HR) during CCO-CPR is lower in individuals with higher cardiorespiratory fitness who have performed moderate aerobic training such as running, cycling, or swimming [12]. In addition, muscle strength [13, 14] and body weight [15] are possible factors affecting exercise intensity during CCO-CPR. Since physical exertion during CC strains the upper part of the body more than the lower part of the body [16], it is likely that physical fitness of the upper body is an important determinant of exercise intensity during CCO-CPR. To date, however, the kinds of physical fitness of the upper body, e.g., endurance, muscle strength, body weight, or other factors, that affect exercise intensity during CCO-CPR have not been fully determined.

Because HR during CCO-CPR has been shown to be a submaximal level [11–15], CC is considered to be a submaximal upper body exercise in a steady rhythm. The unit of load for CC is expressed as work rate [17]. Thus, it is possible that peak work rate of the upper body, which can be increased by upper body training, determines the level of exercise intensity during CCO-CPR because relative work rate during CCO-CPR becomes lower with higher peak work rate of the upper body. The purpose of the present study was to examine the relationship between peak work rate of the upper body and exercise intensity during CCO-CPR. In the present study, we used HR and rating of perceived exertion (RPE) of Borg’s scale as indices of exercise intensity and compared the levels of exercise intensity during CCO-CPR in upper-body-trained individuals, i.e., rugby players and swimmers, with the levels in sedentary individuals. The reason why two groups of the upper-body-trained individuals were recruited was to determine the effect of possible differences in muscle strength and body weight of the upper body on exercise intensity during CCO-CPR based on the assumption that rugby players have more muscle strength and body weight of the upper body.

## Methods

### Subjects

Healthy male university students (*n* = 34) participated in the present study. The subjects included sedentary individuals (group Se, *n* = 12), rugby players (group R, *n* = 11), and swimmers (group Sw, *n* = 11) (Table 1). Subjects in group R and group Sw had more than 4 years of experience in their sports. The subjects in group R participated in field training for 4–6 days per week and in muscular training for 2–6 days per week. The workout sessions in field training and muscular training per week amounted to 8–14 and 3–10 h, respectively. The subjects in group Sw participated in pool training for 3–6 days per week and in muscular training for 0–5 days per week. The workout sessions in pool training and muscular training per week amounted to 6–12 and 0–5 h, respectively. All of the subjects were nonsmokers and had taken a CPR course less than five times before the experiment. The study was conducted in accordance with the Helsinki Declaration and was approved by the Ethics Committee of Chubu University in Kasugai-shi, Aichi, Japan. Voluntary consent for participation in this study under the ethics approval was obtained from all subjects after they were informed of the purpose of the experiment, the procedure, and possible risks.

**Table 1:** Physical characteristics of the subjects

		Sedentary individuals (*n* = 12)	Rugby players (*n* = 11)	Swimmers (*n* = 11)
Age	(years)	20 ± 1	20 ± 1	19 ± 1
Height	(cm)	170.5 ± 6.2	172.0 ± 5.5	171.6 ± 6.0
Weight	(kg)	62.29 ± 8.56^bbb^	84.06 ± 13.17^aaa, ccc^	66.09 ± 8.92^bbb^
Body mass index	(kg·m^−1^·m^−1^)	21.3 ± 2.0^bbb^	28.4 ± 4.0^aaa, ccc^	22.5 ± 2.8^bbb^
Percent body fat	(%)	12.9 ± 4.9^b^	18.0 ± 5.0^c^	13.9 ± 4.7
Whole body muscle mass	(kg)	51.14 ± 4.36^bbb^	64.91 ± 7.19^aaa, ccc^	53.67 ± 5.12^bbb^
Upper body weight	(kg)	36.18 ± 5.20^bbb^	49.48 ± 8.17^aaa, ccc^	38.18 ± 5.68^bbb^
Upper body muscle mass	(kg)	31.30 ± 2.61^bbb^	39.90 ± 4.80^aaa, ccc^	32.63 ± 3.41^bbb^
Peak WR_-AC_	(watt)	108 ± 12^bb, ccc^	139 ± 27^aa^	146 ± 24^aaa^
Peak $\overset{.}{V}$O_2-AC_	(ml·min^−1^)	1940 ± 297^b, ccc^	2441 ± 510^a^	2724 ± 394^aaa^
Relative peak $\overset{.}{V}$O_2-AC_	(ml·min^−1^·kg^−1^)	38.05 ± 5.79^ccc^	37.63 ± 6.67^ccc^	50.82 ± 6.17^aaa, bbb^
Peak ISLE	(kgf)	121 ± 14^b^	152 ± 29^a,c^	118 ± 37^b^

Values are means ± standard deviation

*Peak WR*_*-AC*_ peak work rate during arm-crank exercise, *Peak* $\overset{.}{V}$O_2-AC_ peak oxygen uptake during arm-crank exercise, *Peak ISLE* peak isometric strength of lumbar extension

^a^ *P* < 0.05, ^aa^ *P* < 0.01, and ^aaa^ *P* < 0.001, compared to the value in the group of sedentary individuals

^b^ *P* < 0.05, ^bb^ *P* < 0.01, and ^bbb^ *P* < 0.001, compared to the value in the group of rugby players

^c^ *P* < 0.05, ^cc^ *P* < 0.01, and ^ccc^ *P* < 0.001, respectively, compared to the value in the group of swimmers

### Measurements

Each subject was studied on three different days. On each of the experimental days, the subjects refrained from eating for at least 3 h before the test, from taking caffeine for at least 5 h before the test, and from drinking alcohol and doing heavy exercise for 12 h before the test. All measurements were completed within 24 days.

On the first day, height, weight, and body composition were measured. A multifrequency bioelectrical impedance analyzer (MC-190; TANITA, Tokyo, Japan) was used for measurement of weight and for calculation of body mass index and muscle and fat masses of the four limbs and trunk. Results for body composition obtained by a bioelectrical impedance method using BC-418, also produced by TANITA, have been shown to correlate well with results obtained from dual energy X-ray absorptiometry [18]. According to TANITA, the accuracy of estimates of the composition of each body part produced by MC-190 is equal to that of estimates produced by BC-418 because both use the same system [19]. Thus, estimates from a multifrequency bioelectrical impedance method are considered to be accurate and valid. In the present study, whole body muscle mass, upper body muscle mass, and upper body weight were calculated as the sum of muscle masses of the four limbs and trunk, as the sum of the muscle of the upper limbs and trunk, and as the sum of the muscle and fat masses of the upper limbs and trunk, respectively.

On the second day, peak isometric strength of lumbar extension (Peak ISLE) was determined in a standing posture with lumbar flexion at 30° using a dynamometer (T.K.K.5710C; Takei, Niigata, Japan) because it is assumed that not only back muscles but also muscles of the upper limbs and chest contribute to peak ISLE [20] and indeed because various upper body muscles are active during CC [15, 21]. With a sufficient rest period after peak ISLE measurement, the subjects performed ramp exercise in a sitting position using an arm-crank ergometer (Angio V2; Lode, Groningen, the Netherlands) to determine peak work rate and peak oxygen uptake during arm-crank exercise (Peak WR_-AC_ and Peak $\overset{.}{\mathrm{V}}$O_2-AC_, respectively). The center of the ergometer shaft was positioned at the level of the acromion. After 4 min of 10 watt-warm up, the workload was increased by 10 watts every minute, and rpm was set at 60. The subjects were encouraged to maximize use of their whole upper body muscles during arm-crank exercise according to the method described by Sawka [22]. Arm-crank exercise was terminated when rpm fell below 50 due to exhaustion. Pulmonary oxygen uptake was measured using a respiratory gas analyzer (AE-300S; Minato Medical Science, Osaka, Japan). Peak $\overset{.}{\mathrm{V}}$O_2-AC_ was expressed as a relative term by dividing peak $\overset{.}{\mathrm{V}}$O_2-AC_ by whole body muscle mass (ml·min^−1^·kg^−1^). All measurements were made in a room with the temperature set at a comfortable temperature for each subject (20 ~ 27.5 °C).

After the end of measurements on the first and second days, short (about 3 min) CCO-CPR practice sessions were performed so that the subjects would be able to maintain the prescribed compression rate, compression depth, and recoil and would be able to smoothly answer RPE for the CCO-CPR test to be performed on the third day.

At least 3 days after measurements on the second day, the CCO-CPR test was performed in a room with the temperature set at a comfortable temperature for each subject (21 ~ 26 °C). We used a CPR skill report system consisting of a manikin (CPR501 APPA-Kun Pro; Alexon, Itami, Japan) and a personal computer with dedicated software installed. The same type of manikin has been used previously [23]. The manikin consists of only the thorax portion, and the size is 320 (W) mm × 255 (D) mm × 230 (H) mm. Thorax resistance is provided by the combination of a spring and air. The time course change in CC depth is displayed in real time on the screen of the personal computer, and the data are stored at 200 Hz on the personal computer. The depth-force relationship was measured by the manufacturer in the following way. An aluminum plate was set on the standard compression point of the manikin. The size of the plate was 83 mm × 40 mm. This size is assumed to be equal to the contact area of the hand during actual chest compression. The plate was pushed using a cylinder with a load cell attached to the tip of the cylinder. The depth-force relationship was measured every 10 mm in depth up to 60 mm in depth.

The compression depth-force relationship measured by the manufacturer is linear and is described by the following equation:

1 $$ \mathrm{Compression}\ \mathrm{force}\ \mathrm{required}\ \left(\mathrm{kgf}\right) = 0.6797 \times \mathrm{Compression}\ \mathrm{depth}\ \left(\mathrm{mm}\right) - 2.3067. $$

Equation 1 indicates that the force required to reach a depth of 50 mm is about 32 kgf. This value corresponds to the force required to reach a depth of 50 mm for human males at 60 ± 16 years of age (*n* = 26, 30.5 ± 8.8 kgf) according to the report by Retzer et al. [24].

Each subject rested in a sitting position on the floor for 3 min and then performed 10-min CCO-CPR while kneeling beside a manikin on the floor. This compression time was chosen because the national average time required from the call for an ambulance to the arrival of the ambulance on the scene in Japan as of 2013 is 8.5 min. In the present study, each subject maintained compression depth of more than 50 mm and recoil of less than 5 mm by reference to compression data displayed on the screen and maintained a compression rate of 110 compressions/min with the tempo of a metronome. The mode of CC was determined on the basis of 2010 American Heart Association guidelines [25]. After completing CCO-CPR, the subjects recovered for 6 min in a sitting position on the floor.

An electrocardiogram was obtained at a sampling rate of 1000 Hz using a bioamplifier (Dual BIO Amp; AD Instruments, Bella Vista, Australia). HR was determined from R-R intervals. We calculated the average of HR for 1 min in the middle of the 3-min rest period and the averages of HR every minute during CCO-CPR to use for statistical analysis. RPE during CCO-CPR was determined every minute using Borg’s 15-point scale (6 ~ 20; 9 = very light, 13 = somewhat hard, 17 = very hard) [26]. The subjects were instructed to answer RPE as the sum of strains of local muscles and the cardiorespiratory system. During the recovery period immediately after the end of CCO-CPR, the subjects were asked about the presence or absence of feelings of local arm fatigue and breathlessness.

We conducted an additional experiment in 20 of the 34 subjects (group Se, *n* = 7; group R, *n* = 6; group Sw, *n* = 7). Fourteen subjects did not participate in the additional experiment because some of these subjects refused to participate and other subjects had retired from regular training. The 20 subjects performed both ramp arm-crank exercise and 10-min CCO-CPR. In this experiment, $\overset{.}{\mathrm{V}}$O_2_ during CCO-CPR was measured to estimate the work rate of CCO-CPR from the $\overset{.}{\mathrm{V}}$O_2_-work rate relationship obtained during ramp exercise. Recordings of an electrocardiogram during CCO-CPR and RPE at the 10th minute of CCO-CPR were also conducted.

### Data analysis

The qualities of compression depth and recoil were calculated using the following equations:

$$ \mathrm{C}\mathrm{C}\ \mathrm{quality}\hbox{-} \mathrm{depth}\ \left(\%\right) = \frac{\mathrm{Number}\ \mathrm{of}\ \mathrm{compressions}\ \mathrm{a}\mathrm{t}\ \mathrm{a}\ \mathrm{depth}\ \mathrm{of}\ \mathrm{more}\ \mathrm{t}\mathrm{han}\ 50\ \mathrm{mm}\ \mathrm{per}\ \mathrm{minute}}{\mathrm{Number}\ \mathrm{of}\ \mathrm{t}\mathrm{otal}\ \mathrm{compressions}\ \mathrm{per}\ \mathrm{minute}} \times 100 $$

$$ \mathrm{C}\mathrm{C}\ \mathrm{quality}\hbox{-} \mathrm{recoil}\ \left(\%\right) = \frac{\mathrm{Number}\ \mathrm{of}\ \mathrm{recoils}\ \mathrm{a}\mathrm{t}\ \mathrm{a}\ \mathrm{depth}\ \mathrm{less}\ \mathrm{t}\mathrm{han}\ 5\ \mathrm{mm}\ \mathrm{per}\ \mathrm{minute}}{\mathrm{Number}\ \mathrm{of}\ \mathrm{t}\mathrm{otal}\ \mathrm{recoils}\ \mathrm{per}\ \mathrm{minute}} \times 100. $$

Work rate of every compression was calculated as follows:

- Work rate of compression (watt)=Work of compression (J)^※1^ / Times taken for compression (sec);
- ※1⋯Work of compression (J)= Compression force (N)^※2^ × Compression depth (m);
- ※2⋯Compression force (N)= Compression force (kgf) / 0.10197.

Time taken for compression was calculated from time series data on compression depth. We calculated the averages of work rate of chest compression every minute (WR_-CC_) to use for statistical analysis.

Since CC and arm-crank exercise are different types of movement, calculation of relative work rate of CCO-CPR as the ratio of WR_-CC_ to peak WR_-AC_ may be invalid. Thus, as mentioned above, we estimated WR_-CC_ using the $\overset{.}{\mathrm{V}}$O_2_-work rate relationship during ramp arm-crank exercise. The $\overset{.}{\mathrm{V}}$O_2_-work rate relationship was expressed by a quadratic function rather than a linear function because $\overset{.}{\mathrm{V}}$O_2_ during ramp arm-crank exercise increases curvilinearly with increase in work rate possibly due to excessive body movement at a high work rate [22]. Then, the average of $\overset{.}{\mathrm{V}}$O_2_ for the last 1 min of CCO-CPR was calculated and substituted into the equation of the $\overset{.}{\mathrm{V}}$O_2_-work rate relationship to estimate the work rate of CCO-CPR (estimated WR_-CC_). Estimated relative WR_-CC_ was determined as the ratio of estimated WR_-CC_ to peak WR_-AC_.

### Statistical analysis

Data for physical characteristics in the three groups were analyzed through a one-way analysis of variance (ANOVA). Data for HR, RPE, compression rate, CC quality-depth, CC quality-recoil, and WR_-CC_ in the CCO-CPR test were analyzed through a two-way repeated-measures ANOVA with group category and time within an experimental trial. Tukey’s honestly significant difference test was used as a post hoc test. Multiple linear regression analysis with the stepwise method was carried out to assess potential relationships of upper body weight, peak ISLE, peak WR_-AC_, and relative peak $\overset{.}{\mathrm{V}}$O_2-AC_, with HR and RPE during CCO-CPR. Pearson’s correlation analysis was used to assess relationships of estimated WR_-CC_ and estimated relative WR_-CC_ with HR and RPE during CCO-CPR. Pearson’s correlation analysis was also used to assess the relationship between upper body muscle mass and peak ISLE. Partial correlation analysis was used to assess the relationship between peak ISLE and peak WR_-AC_ when upper body muscle mass was included as a control variable. A value of *P* < 0.05 was regarded as statistically significant. All data are presented as means ± standard deviation.

## Results

### Physical characteristics in the three groups (Table 1)

Group R showed significantly larger values in weight (*P* < 0.001), body mass index (*P* < 0.001), whole body muscle mass (*P* < 0.001), upper body weight (*P* < 0.001), upper body muscle mass (*P* < 0.001), and peak ISLE (*P* < 0.05) than those in the other two groups. On the other hand, group Sw showed a significantly larger value in relative peak $\overset{.}{\mathrm{V}}$O_2-AC_ than those in the other two groups (*P* < 0.001). Peak WR_-AC_ was significantly larger in group R and group Sw than in group Se (group R, *P* < 0.01 and group Sw, *P* < 0.001). There was a significant positive relationship between upper body muscle mass and peak ISLE (*r* = 0.72, *P* < 0.001). The relationship between peak ISLE and peak WR_-AC_ was not significant.

### CCO-CPR

**Fig. 1:**
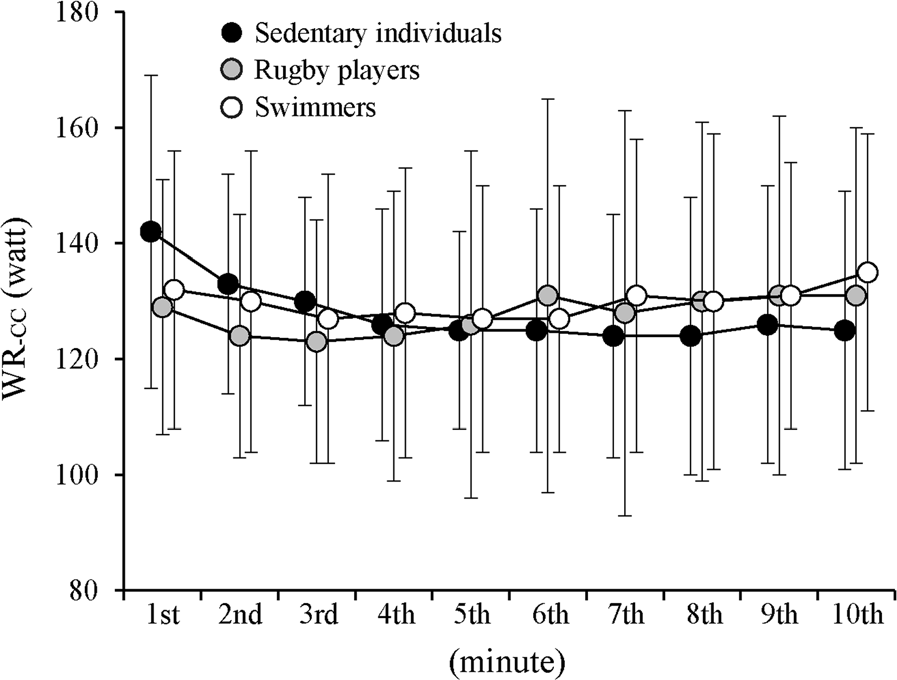
Comparisons of changes in work rate during 10-min chest compressions between groups. *WR*_*-CC*_ work rate of chest compression

Compression rate, CC quality-depth, and CC quality-recoil from the first minute to the last minute of CCO-CPR for all subjects ranged from 108 to 119 compressions/min, from 68 to 100 %, and from 93 to 100 %, respectively. There was no main effect of group or time, and there was no interaction in the three parameters. Overall averages (34 subjects × 10 time points) of compression rate, CC quality-depth, and CC quality-recoil were 110 ± 1 compressions/min, 99 ± 4 %, and 99 ± 1 %, respectively, indicating good qualities of compression depth and recoil throughout the 10-min CCO-CPR. Figure 1 shows WR_-CC_ during CCO-CPR. There was no main effect of group or time, and there was no interaction. The averages of WR_-CC_ during CCO-CPR ranged from 123 to 143 watts.

**Fig. 2:**
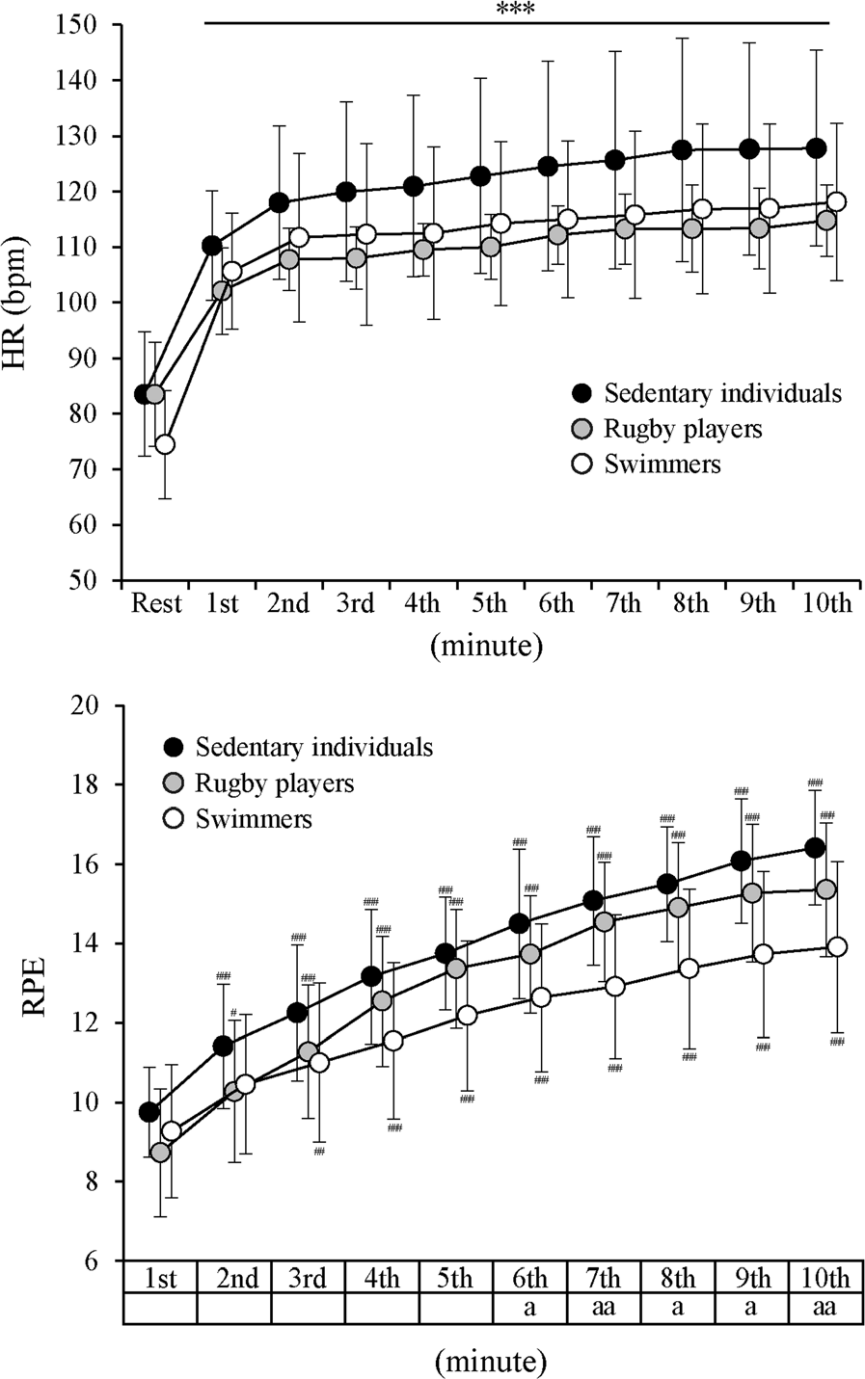
Comparisons of changes in heart rate and rating of perceived exertion during 10-min chest compression between groups. *HR* heart rate, *RPE* rating of perceived exertion. *P < 0.05, **P < 0.01, and ***P < 0.001, compared with the value at rest for HR. ^#^ *P* < 0.05, ^##^ *P* < 0.01, and ^###^ *P* < 0.001, compared with the value at the first minute of chest compression for RPE. a *P* < 0.05 and aa *P* < 0.01, between values in the group of sedentary individuals and the group of swimmers

Figure 2 shows HR and RPE responses during CCO-CPR. HR increased with time up to 127.8 ± 17.6, 114.8 ± 6.5, and 118.1 ± 14.2 bpm at the 10th minute in group Se, group R, and group Sw, respectively. Although HR level tended to be higher in group Se than in the other two groups, there was no significant difference between the three groups. Only a significant main effect for time was observed. On the other hand, RPE increased with time up to 16.4 ± 1.4, 15.4 ± 1.7, and 13.9 ± 2.2 at the 10th minute in group Se, group R, and group Sw, respectively. RPEs from the sixth minute to last minute in group Sw were significantly lower than those in group Se (*P* < 0.05). Multiple linear regression analysis showed that HR at all time points of CCO-CPR can be explained only by peak WR_-AC_, and the analysis showed that RPE level at the 2nd minute and from the 6th minute to the 10th minute of CCO-CPR can also be explained only by peak WR_-AC_. Standardized partial regression coefficients ranged from −0.421 to −0.482 for HR (*P* < 0.01) and from −0.340 to −0.463 for RPE (*P* < 0.05). RPE level at the first min was determined by upper body weight, but none of the factors could determine RPE levels from the third to the fifth min.

There was a feeling of breathlessness in 5 of the 12 subjects in group Se (41.7 %), in 5 of the 11 subjects in group R (45.5 %), and in 5 of the 11 subjects in group Sw (45.5 %). On the other hand, there was a feeling of fatigue of the arm in 11 of the 12 subjects in group Se (91.7 %), in 10 of the 11 subjects in group R, (90.9 %), and in 5 of the 11 subjects in group Sw (45.5 %).

**Fig. 3:**
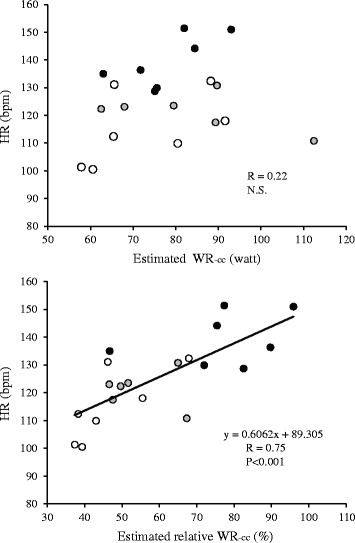
Relationship between estimated work rate and heart rate during CCO-CPR. *HR* heart rate, *WR*_*-CC*_ work rate of chest compression. *Black*, *grey*, and *white circles* represent sedentary individuals, rugby players, and swimmers, respectively

In the CCO-CPR test of the additional experiment, values of $\overset{.}{\mathrm{V}}$O_2_ at rest in group Se, group R, and group Sw were 280 ± 39, 345 ± 62, and 300 ± 32 ml·min^−1^, respectively. $\overset{.}{\mathrm{V}}$O_2_ increased with time, reaching 1258 ± 162, 1461 ± 260, and 1231 ± 109 ml·min^−1^ at the 10th minute of CCO-CPR in group Se, group R, and group Sw, respectively (Table 2). Although there was no significant difference in estimated WR_-CC_ among the three groups, estimated relative WR_-CC_ was significantly lower in group R and group Sw than that in group Se (group R, *P* < 0.05 and group Sw, *P* < 0.001) (Table 2). Estimated relative WR_-CC_ ranged from 37 to 96 % across all subjects. Figure 3 shows the relationship between estimated relative WR_-CC_ and HR at the 10th minute. There was a strong positive linear relationship between the two variables (*R* = 0.75, *P* < 0.001), although HR at the 10th minute was not significantly related to estimated WR_-CC_ itself. On the other hand, there was no significant relationship between estimated WR_-CC_ and RPE or between estimated relative WR_-CC_ and RPE.

**Table 2:** Results in the ramp arm-crank exercise and CCO-CPR of the additional experiment

			Sedentary individuals (*n* = 7)	Rugby players (*n* = 6)	Swimmers (*n* = 7)
Ramp exercise	Peak WR_-AC_	(watt)	104 ± 17^bbb, ccc^	153 ± 22^aaa^	158 ± 19^aaa^
	Peak $\overset{.}{V}$O_2-AC_	(ml·min^−1^)	1753 ± 234^bbb, ccc^	2615 ± 254^aaa^	2518 ± 298^aaa^
CCO-CPR	$\overset{.}{V}$O_2_ at the 10th min	(ml·min^−1^)	1258 ± 162	1461 ± 260	1231 ± 109
	Ratio of $\overset{.}{V}$O_2_ at the 10th min to Peak $\overset{.}{V}$O_2-AC_	(%)	73 ± 13^b, ccc^	56 ± 7^a^	49 ± 7^aaa^
	HR at the 10th min	(bpm)	140 ± 9^b, ccc^	121 ± 7^a^	115 ± 13^aaa^
	RPE at the 10th min		15.3 ± 1.6	14.2 ± 1.7	13.9 ± 1.2
	Estimated WR_-CC_	(watt)	78 ± 10	84 ± 18	73 ± 14
	Estimated Relative WR_-CC_	(%)	77 ± 16^b, ccc^	55 ± 9^a^	47 ± 11^aaa^

Values are means ± standard deviation

*Peak WR*_*-AC*_ peak work rate during arm-crank exercise, *Peak* $\overset{.}{V}$O_*2-AC*_ peak oxygen uptake during arm-crank exercise, *CCO-CPR* chest-compression-only cardiopulmonary resuscitation, *WR*_*-CC*_ work rate of chest compression

^a^ *P* < 0.05, ^aa^ *P* < 0.01, and ^aaa^ *P* < 0.001, compared to the value in the group of sedentary individuals

^b^ *P* < 0.05, ^bb^ *P* < 0.01, and ^bbb^ *P* < 0.001, compared to the value in the group of rugby players

^c^ *P* < 0.05, ^cc^ *P* < 0.01, and ^ccc^ *P* < 0.001, compared to the value in the group of swimmers

## Discussion

### Comparison of levels of physical fitness in the three groups

Upper body muscle mass was closely related to peak ISLE, suggesting that peak ISLE reflects muscle strength of the upper body and that subjects in group R could exert higher peak ISLE due to greater mass of upper body muscles. However, partial correlation analysis showed that peak WR_-AC_ was not related to peak ISLE in part because peak ISLE was significantly lower in group Sw than that in group R regardless of the fact that peak WR_-AC_ in group Sw and that in group R were almost the same. It is possible that subjects in group Sw could have exerted force during arm-crank exercise that was not properly reflected by peak ISLE because the subjects were encouraged to maximize use of their whole upper body muscles and because more muscle mass might have been recruited in subjects in group Sw possibly due to the similarity of movement patterns for swimming stroke (especially crawl stroke) and arm-crank exercise.

### Determinants of degree of exercise intensity during chest compression

The unit of load is expressed as work rate in CCO-CPR [17]. Badaki-Makun et al. reported that peak work rates during 10-min CCO-CPR were 144.1 watts in a child manikin and 166.5 watts in an adult manikin [17]. In the present study, on the other hand, the peak value of average WR_-CC_ was 143 watts, corresponding to the value during CCO-CPR using a child manikin [17].

The unit of load of arm-crank exercise is also expressed as work rate. In the present study, only peak WR_-AC_ was a determinant of HR at all time points of CCO-CPR. Although arm-crank exercise and CC are different types of exercise, both are upper body exercises in a steady rhythm. Thus, it is possible that HR becomes lower with higher peak WR_-AC_ due to a reduction of relative work rate during CCO-CPR. In fact, subjects with lower estimated relative WR_-CC_ showed lower HR. In the present study, RPE level in the latter 5 min of CCO-CPR was also determined only by peak WR_-AC_. However, RPE in the former 5 min of CCO-CPR except RPE at the second minute of CCO-CPR could not be determined by peak WR_-AC_. In addition, there was no significant relationship between estimated relative WR_-CC_ and RPE level. Thus, the results suggested that RPE level is not directly related to peak WR_-AC_. Pierce et al. [27] examined local muscular and cardiopulmonary strains separately by using Borg’s RPE scale during 10-min conventional CPR. They found that the RPE for local muscular strains was higher than the RPE for cardiopulmonary strain. Thus, RPE during CCO-CPR might have been greatly influenced by local muscle strain in the present study, although we instructed the subjects to give overall RPE including strains of local muscle and cardiopulmonary systems. In support of this possibility, RPE levels from the sixth minute to the last minute were lower in group Sw, in which fewer subjects reported feeling arm fatigue immediately after the end of CCO-CPR. If RPE during CCO-CPR is determined mainly by local muscle fatigue, no significant difference in RPE between the three groups in the former 5 min of CCO-CPR might have been due to little difference in local muscle fatigue.

Muscle strength [13, 14] and weight [15] are possible factors affecting exercise intensity during CCO-CPR. In the present study, although upper body weight or muscle strength as evaluated from peak ISLE was not an explanatory variable for HR and RPE during CCO-CPR, higher muscle strength will serve to increase peak WR_-AC_. In addition, large upper body weight will enable performance of CC with less muscle strength due to the action of gravity. Thus, it is likely that muscle strength and upper body weight are remote causes of a decrease in exercise intensity during CCO-CPR.

### Implications and limitations

Previous reports have pointed out the need for a minimum level of physical conditioning in CPR providers because a higher level of physical fitness has a positive impact on the quality of CCO-CPR [12–14]. In the present study, peak WR_-AC_, which determines HR during CCO-CPR, was larger in group R and group Sw than that in group Se. In addition, group Sw showed a low RPE level during CCO-CPR compared to that in group Se. These findings show the importance of physical conditioning of the upper body for the quality of CCO-CPR. However, although upper body weight and peak ISLE were lower in group Sw than those in group R, the subjects in group Sw could perform CCO-CPR with levels of HR and RPE similar to those in subjects in group R, indicating that training that enhances muscle strength and increases body weight of the upper body is not necessarily required for performance of CCO-CPR with a low level of exercise intensity. Our findings will eventually contribute to education and training of single-rescuer bystander CPR for potential CPR providers such as citizens and all health professionals involved in pre- and in-hospital emergency medicine and critical care. Furthermore, we believe that our findings are important in terms of life-saving for elderly people in an aging society considering that an issue in an aging society is an increase in the number of elderly patients with out-of-hospital cardiac arrest [5] and that CCO-CPR is a useful tool for elderly patients [1, 5]. In the present study, we focused on single-rescuer bystander CPR. However, it should be noted that participation of more than one bystander and changes of their roles would lead to an increase in the rate of success of CPR through maintenance of the quality of chest compressions during CCO-CPR [28].

A major limitation of this study is that the results cannot be extended to CCO-CPR by humans with higher chest resistance. In the present study, a force of about 32 kgf was required for 50 mm depth compression. In this situation, estimated WR_-CC_ was a submaximal level ranging from 37 to 96 % of peak WR_-AC_. However, Aelen et al. [29] demonstrated that compression force required for a depth of 50 mm ranges from 14.0 to 96.4 kgf in humans. In the case of compressions of a stiffer chest, exercise intensity will increase in the maximal or supramaximal level, causing difficulty in continuation of CCO-CPR. Therefore, endurance capacity for sustaining a higher peak work rate of the upper body may be needed during CCO-CPR for a stiffer chest. Further study is needed to clarify what kind of this possibility.

## Conclusions

HR during CCO-CPR is lower in individuals who have a higher peak work rate of the upper body due to reduction of relative work rate during CCO-CPR. Since upper-body-trained individuals have the potential not only to exert higher peak WR_-AC_ but also to execute CCO-CPR with a low level of RPE, upper body training is useful for reduction of exercise intensity during CCO-CPR. However, training that enhances muscle strength and increases body weight of the upper body is not necessarily required for performance of CCO-CPR with a low level of exercise intensity.

## Abbreviations

CC: chest compression
CCO-CPR: chest-compression-only cardiopulmonary resuscitation
CPR: cardiopulmonary resuscitation
Group Se: group of sedentary individuals
Group R: group of rugby players
Group Sw: group of swimmers
HR: heart rate
Peak ISLE: peak isometric strength of lumbar extension
Peak $\overset{.}{\mathrm{V}}$O_2-AC_: peak oxygen uptake during arm-crank exercise
Peak WR_-AC_: peak work rate during arm-crank exercise
RPE: rating of perceived exertion
WR_-CC_: work rate of chest compression
